# MicroRNA-206 is involved in the pathogenesis of ulcerative colitis via regulation of adenosine A3 receptor

**DOI:** 10.18632/oncotarget.13525

**Published:** 2016-11-23

**Authors:** Weiyun Wu, Yanting He, Xiao Feng, Shicai Ye, Hao Wang, Wenkai Tan, Caiyuan Yu, Juxiang Hu, Rong Zheng, Yu Zhou

**Affiliations:** ^1^ Department of Gastroenterology, Affiliated Hospital of Guangdong Medical University, Zhanjiang 524001, China

**Keywords:** miRNA-206, ulcerative colitis, adenosine A3 receptor, NF-κB

## Abstract

Increasing evidence suggests that miRNAs are widely dysregulated in ulcerative colitis (UC), potentially affecting UC pathogenesis, diagnosis, and therapy. microRNA (miR) −206 has been reported to be upregulated in UC; however, its function and role in UC remain unknown. Here, we elucidate the function of miR-206 in the pathogenesis of UC. In patients with active-UC, miR-206 and adenosine A3 receptor (A3AR) levels were significantly upregulated and downregulated, respectively, and were inversely correlated. A3AR was expressed in the colon mucosa (particularly in colon epithelial-cell membranes). In HT-29 cells, miR-206 downregulated A3AR mRNA/protein expression by directly targeting the A3AR 3′-UTR; miR-206 overexpression and knockdown respectively increased and decreased TNF-α-induced nuclear NF-κB/p65, p-IκB-α, IKKα, p-IKKα and IL-8/IL-1β secretion. However, A3AR-siRNA reversed the miR-206 inhibitory effect. Furthermore, miR-206 increased dextran sodium sulphate-induced colitis severity (i.e., increased bodyweight loss, DAI score, colon shrinkage, and MPO activity), which was partially ameliorated by miR-206-antagomir treatment. miR-206-agomir treatment potently suppressed A3AR expression and increased NF-κB signalling and downstream cytokine (TNF-α/IL-8/IL-1β) expression in the mouse colon, in contrast to miR-206-antagomir administration. Taken together, our results demonstrated that miR-206 has a proinflammatory role in UC by downregulating A3AR expression and activating NF-κB signalling.

## INTRODUCTION

Ulcerative colitis (UC) is a chronic and recurrent inflammatory bowel disease (IBD) that affects the rectum and colon. UC is characterised by mucosal ulceration, which generates symptoms of abdominal pain and bloody diarrhoea [1]. In China, economic development and the associated changes in diet and lifestyle have been accompanied by reports of increasing UC incidence over the last 20 years [2]. IBD pathogenesis is incompletely understood, however, IBD is hypothesised to result from an inappropriate immune response to gut microbiota, facilitated by both patient genetic susceptibility and environmental factors [3]. In recent decades, new insights into the functions of microRNAs (miRNAs) during UC pathogenesis have emerged, with a concomitant enhancement of interest in the potential of miRNAs as UC biomarkers and therapeutic targets [4, 5].

miRNAs comprise 18–25-nucleotide-long noncoding RNAs. Mature miRNAs combine with the RNA-induced silencing complex and then completely or incompletely bind to sequences in the 3′-untranslated region (3′-UTR) of target mRNAs. miRNA binding results in target-mRNA degradation or translation inhibition, thus regulating the expression of multiple protein-coding genes [6]. miRNAs function as key regulators of various pathophysiological processes- including cell proliferation, differentiation, apoptosis, cancer, autoimmunity, and inflammation [7, 8]. Increasing evidence suggests that miRNAs are widely dysregulated in UC, potentially impactingUC pathogenesis, diagnosis, and therapy [9]. The miRNA-UC relationship was first reported in 2008 by Wu et al. [10] who found that active UC was associated with the differential expression of 11 miRNAs. Among these, miR-192 showed reduced expression in UC and was demonstrated to target macrophage-inflammatory peptide-2α and downregulate its expression. Subsequent reports have indicated that miRNAs might participate in UC pathogenesis by regulating downstream target genes. For example, Chen et al. [11] found that miR-19a and tumour necrosis factor-α (TNF-α) levels are markedly downregulated and upregulated, respectively, in active human UC and dextran sodium sulphate (DSS)-induced mouse experimental colitis; that miR-19a directly regulates TNF-α; and that miR-19a-inhibitor treatment markedly elevates the expression of the inflammatory factors TNF-α, interleukin-8 (IL-8), and granulocyte-macrophage colony-stimulating factor. Bian et al. [12] reported that miR-150 elevation contributed to colonic epithelial disruption through c-Myb targeting in DSS-induced colitis in mouse. In addition, Feng et al. [13] reported that miR-126 overexpression enhanced UC inflammatory activity by downregulating the expression of Iκ-Bα, a key inhibitor of the NF-κB signalling pathways. These studies have revealed that miRNAs participate in UC pathogenesis by either impairing the intestinal epithelial barrier or regulating inflammatory mediators. Furthermore, miR-206 has been reported to be upregulated in UC [14], although its function in general and as related to UC remains unknown.

A3 adenosine receptor (A3AR) is a subtype of the adenosine-receptor family. A3AR activation inhibits adenylyl cyclase and cAMP formation through the Gi protein [15]. A3AR has been reported to play a protective role in certain pathophysiological processes such as rheumatoid arthritis, myocardial ischemia/reperfusion injury, and colonic inflammation [16–18]. However, A3AR protein expression was shown to be decreased in colorectal mucosal epithelial cells of patients with UC and in colitis animal models [19, 20]. Conversely, A3AR activation could reduce colonic inflammation: Mabley et al. [21] found that the A3AR-agonist IB-MECA protected mice with DSS-induced colitis against inflammatory cell infiltration and damage owing to colitis and attenuated the increase in colon inflammatory cytokine and chemokine levels. In addition, Guzman et al. [22] found that in a rat chronic model of 2,4,6-trinitrobenzene sulphonic acid -induced colitis, oral IB-MECA prevented dysregulated expression of 92% of the colitis-induced genes, limited histopathological gut injury and weight loss, and suppressed free-radical elevation in *ex vivo* inflamed guts. Previously, we found that A3AR was expressed in the human colonic epithelial cell line HT-29 and that its activation exerted an anti-inflammatory effect by inhibiting NF-κB signalling pathways, which resulted in the downstream inhibition of IL-8 and IL-1β expression [18]. We also obtained similar results in the murine DSS-colitis model *in vivo* [23].

Here, we investigated the mechanism by which miR-206 stimulates colonic inflammation. Bioinformatics analysis identified A3AR as a putative miR-206 target. We found miR-206 was upregulated but A3AR mRNA and protein were downregulated in UC, and that miR-206 markedly reduced A3AR mRNA/protein expression in HT-29 cells by directly targeting the A3AR 3′-UTR. Moreover, miR-206 overexpression and knockdown in HT-29 cells respectively increased and decreased TNF-α-induced nuclear NF-κB p65 expression and IL-8/IL-1β secretion. Notably, the miR-206-inhibitory effect could be reversed by A3AR-siRNA. Similar results were obtained using DSS-colitis mice. Our findings therefore provide insights into the proinflammatory function of miR-206 in colonic inflammation via A3AR downregulation.

## RESULTS

### Expression and correlation of miR-206 and A3AR in UC tissue

**Figure 1:**
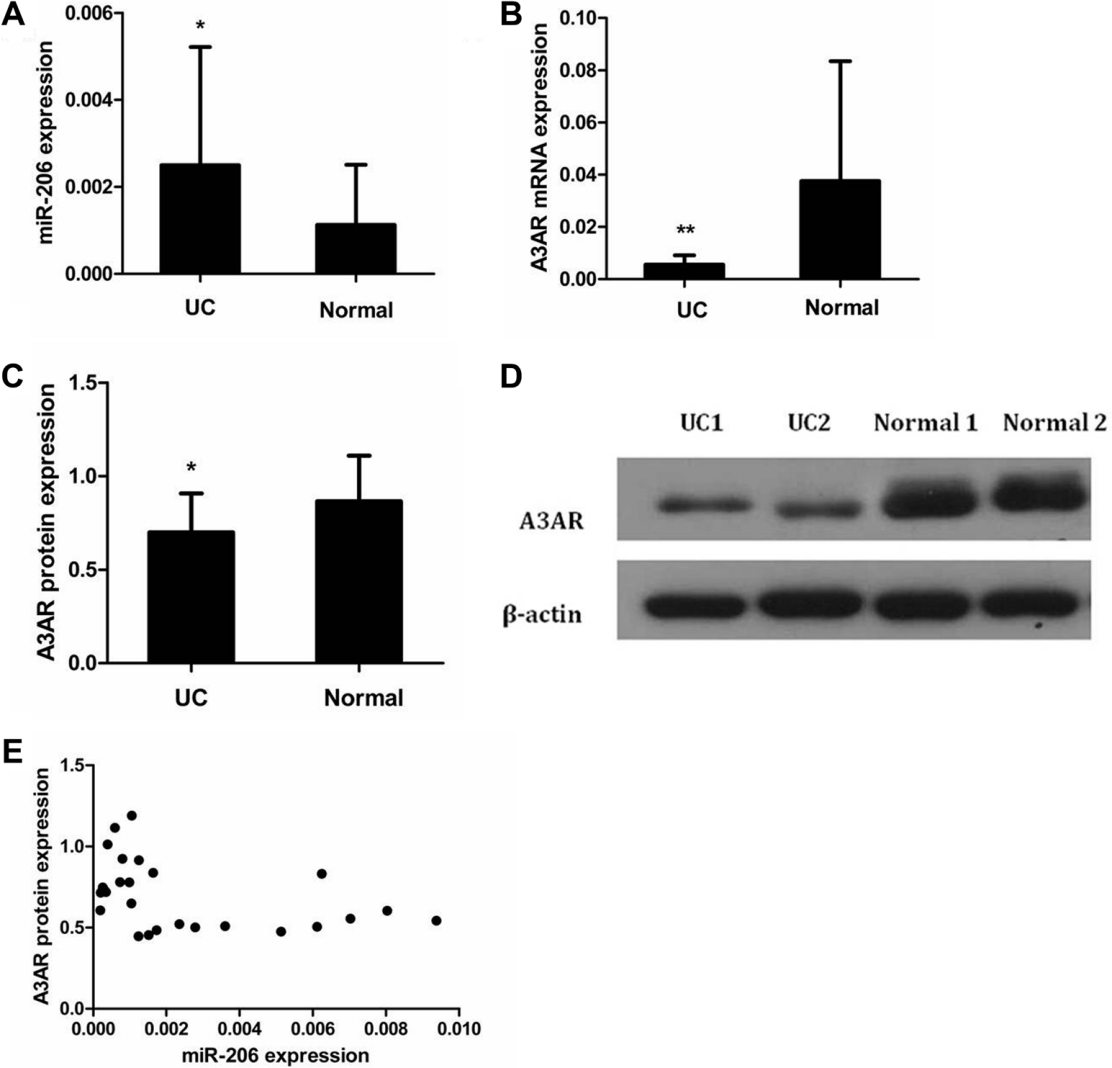
Expression and correlation of miR-206 and A3AR in UC tissues Expression in UC and normal tissues of (**A**) miR-206, (**B**) A3AR mRNA, and (**C**) A3AR protein. (**D**) Representative western blotting showing A3AR protein expression in UC and normal tissues. Data are presented as means ± SD of 3 independent experiments. *P < 0.05; **P < 0.01. (**E**) Correlation of miR-206 and A3AR expression (Pearson correlation *r* = −0.404, *P* < 0.05).

We used quantitative reverse transcription-polymerase chain reaction (qRT-PCR) and western blotting to examine miR-206 and A3AR expression in colonic samples from patients with active-UC and healthy controls. miR-206 levels were significantly higher in active-UC tissues than those in control tissues (*P* < 0.05; Figure 1A). In contrast, A3AR mRNA and protein levels were significantly lower in active-UC tissues than those in normal tissues (*P* < 0.05; Figure 1B–1D). Furthermore, in UC tissues, miR-206 level and A3AR protein expression were inversely correlated (Pearson's *r* = −0.404, *P* < 0.05; Figure 1E).

### A3AR localisation in human colonic tissues

**Figure 2:**
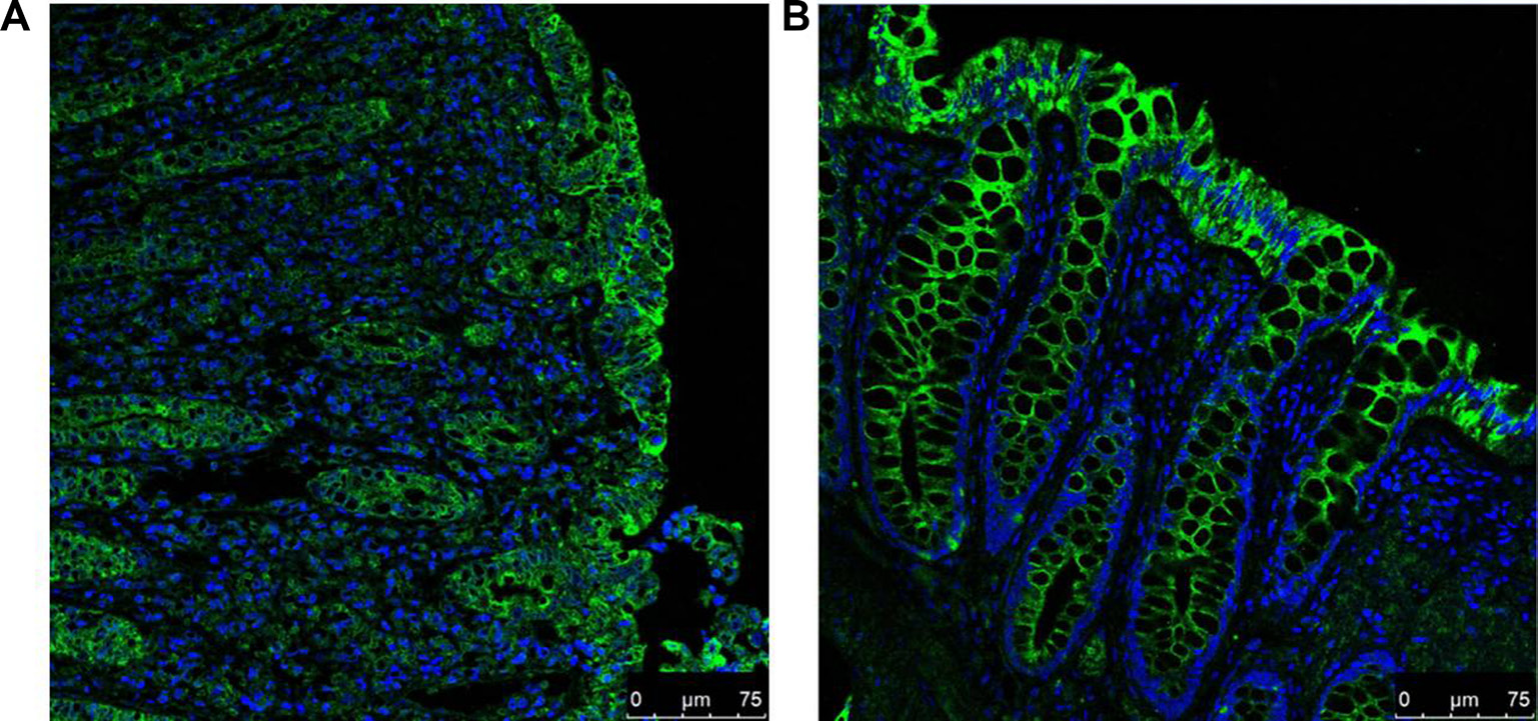
IF staining for A3AR (FITC; green) and counterstaining of nuclei (DAPI; blue) in UC (A) and normal (B) colonic tissues A3AR is observed mainly in the colonic mucosal epithelial-cell membrane. A3AR green fluorescence in UC is weaker than that in normal tissue (magnification 400×).

To localise A3AR expression in human colonic tissues, we performed immunofluorescence (IF) staining on paraffin sections of colon biopsies. A strong green-florescence signal representing A3AR appeared mainly in the epithelial cells of UC and normal colonic mucosa and was concentrated in the cell membrane (Figure 2), consistent with previous reports that A3AR is a transmembrane receptor. A3AR staining in active-UC tissues was weaker than that in normal colonic mucosa.

### miR-206 downregulates A3AR expression

**Figure 3:**
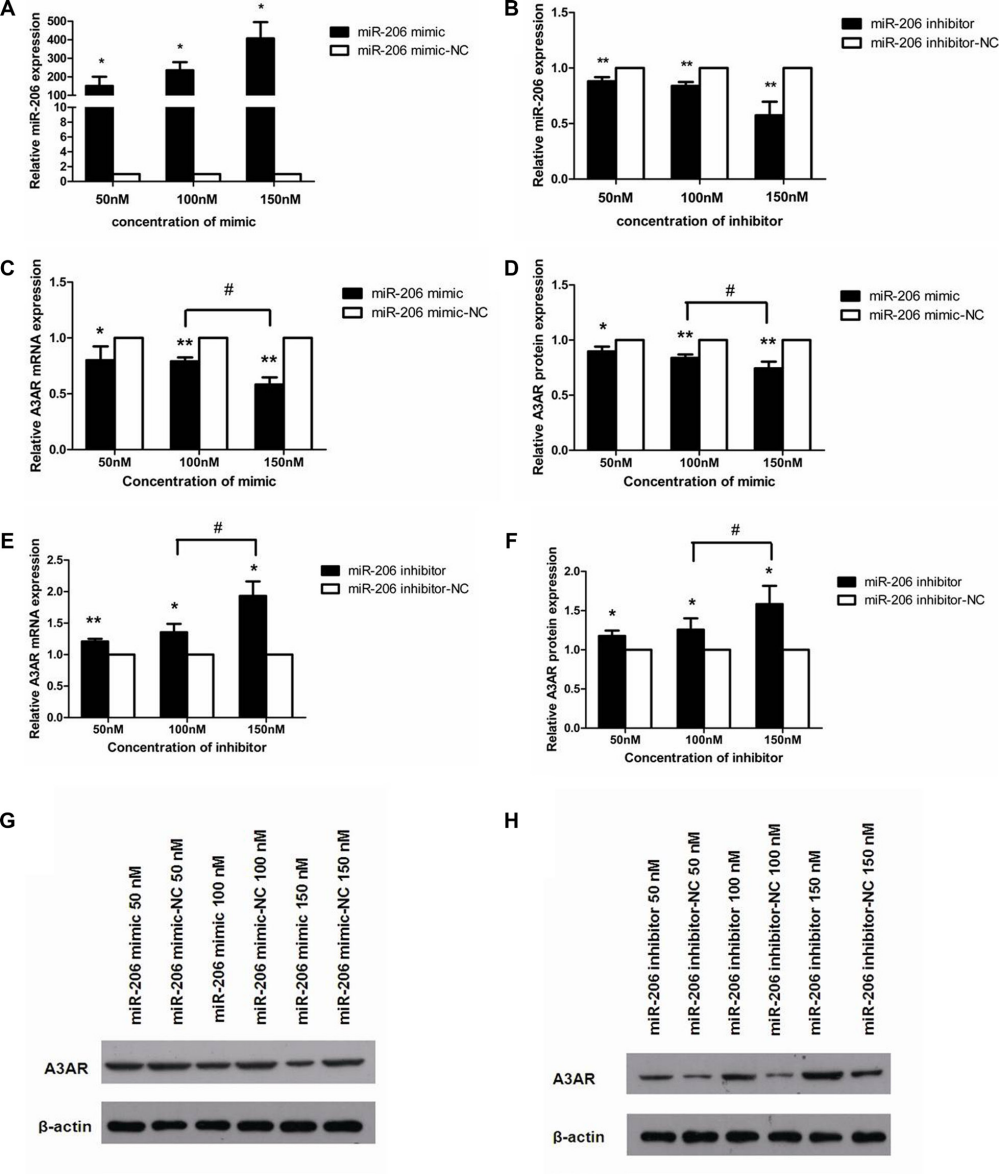
miR-206 downregulated A3AR expression (**A**, **B**) miR-206 expression was increased and decreased in HT-29 cells after transfection with miR-206 mimic and miR-206 inhibitor, respectively, as compared with the expression in cells transfected with the corresponding NCs. (**C**, **D**) A3AR mRNA and protein expression was significantly decreased in a concentration-dependent manner in HT-29 cells transfected with miR-206 mimic, as compared with levels in the corresponding NC-transfected cells. (**E**, **F**) A3AR mRNA and protein expression was significantly increased in a concentration-dependent manner in HT-29 cells transfected with miR-206 inhibitor (relative to NC). (**G**, **H**) Representative western blotting showing A3AR protein expression following various transfections. Results are expressed as fold-change relative to NC and presented as means ± SD of 3 independent experiments. *P < 0.05 and **P < 0.01, compared with corresponding NC; ^#^*P* < 0.05, between indicated groups.

A3AR was identified as a potential miR-206 target by using bioinformatics tools (TargetScan and miRanda). Our *in vivo* data showed that high miR-206 expression correlated with low A3AR protein levels; thus, we further studied this correlation *in vitro* by using HT-29 cells. To test whether miR-206 directly regulates A3AR expression, we transfected HT-29 cells with various concentrations of miR-206 mimics/inhibitors and examined A3AR mRNA and protein levels at 48 and 72 h post-transfection, respectively. qRT-PCR results showed that miR-206 expression was significantly higher in HT-29 cells transfected with the miR-206 mimic than that in cells transfected with the miR-206 mimic-NC (*P* < 0.05; Figure 3A). Furthermore, in cells transfected with the miR-206 mimic, A3AR mRNA and protein levels were significantly downregulated in a concentration-dependent manner (*P* < 0.05; Figure 3C, 3D, and 3G). Incontrast, relative to the corresponding NC group, HT-29 cells transfected with miR-206 inhibitor exhibited significantly lower miR-206 levels (*P* < 0.01; Figure 3B) and showed a significant concentration-dependent increase in both A3AR mRNA and protein expression (*P* < 0.05; Figure 3E, 3F, and 3H). In subsequent experiments, transfections were performed using only the highest concentration (150 nM) of miR-206 mimic/inhibitor/NC. Our results indicated that miR-206 downregulated A3AR expression.

### miR-206 directly targets the A3AR 3′-UTR

**Figure 4:**
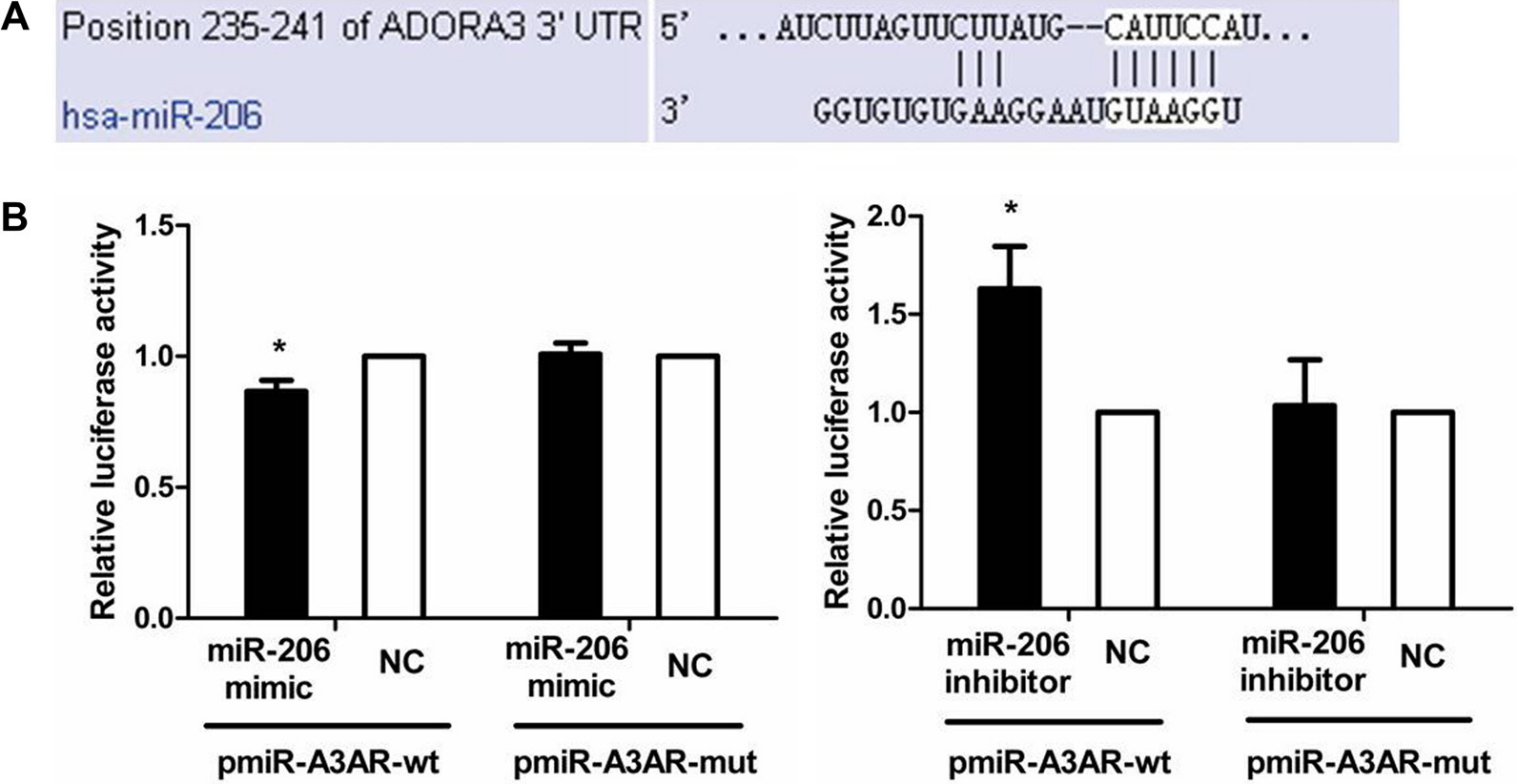
miR-206 targeting of A3AR 3′-UTR (**A**) The miR-206 target site in the 3′-UTR of A3AR mRNA predicted by TargetScan. (**B**) Dual-luciferase reporter assay. HT-29 cells were cotransfected with pmiR-A3AR-wt (or pmiR-A3AR-mut) and miR-206 mimic (or miR-206 inhibitor). Luciferase activity of pmiR-A3AR-wt was significantly decreased and increased by miR-206 mimic and miR-206 inhibitor, respectively, relative to corresponding NCs; however, pmiR-A3AR-mut luciferase activity was unaffected by miR-206 mimic or inhibitor. Results are expressed as fold-change relative to NC and presented as means ± SD of 3 independent experiments. *P < 0.05, compared with NC group.

We performed dual-luciferase reporter assays to investigate whether miR-206 targets the A3AR 3′-UTR (Figure 4). As compared with cotransfection with NC, cotransfection of pmiR-A3AR-wt with the miR-206 mimic resulted in significantly diminished luciferase activity in HT-29 cells (*P* < 0.05), whereas cotransfection of pmiR-A3AR-wt with miR-206 inhibitor led to increased luciferase activity (*P* < 0.05). However, luciferase activity remained unchanged in cells cotransfected with the miR-206 mimic or inhibitor in conjunction with thepmiR-A3AR-mut. These results suggested that A3AR was a direct target of miR-206 in HT-29 cells.

### miR-206 stimulates NF-κB activation and increased IL-8/IL-1β expression in TNF-α-treated cells

**Figure 5:**
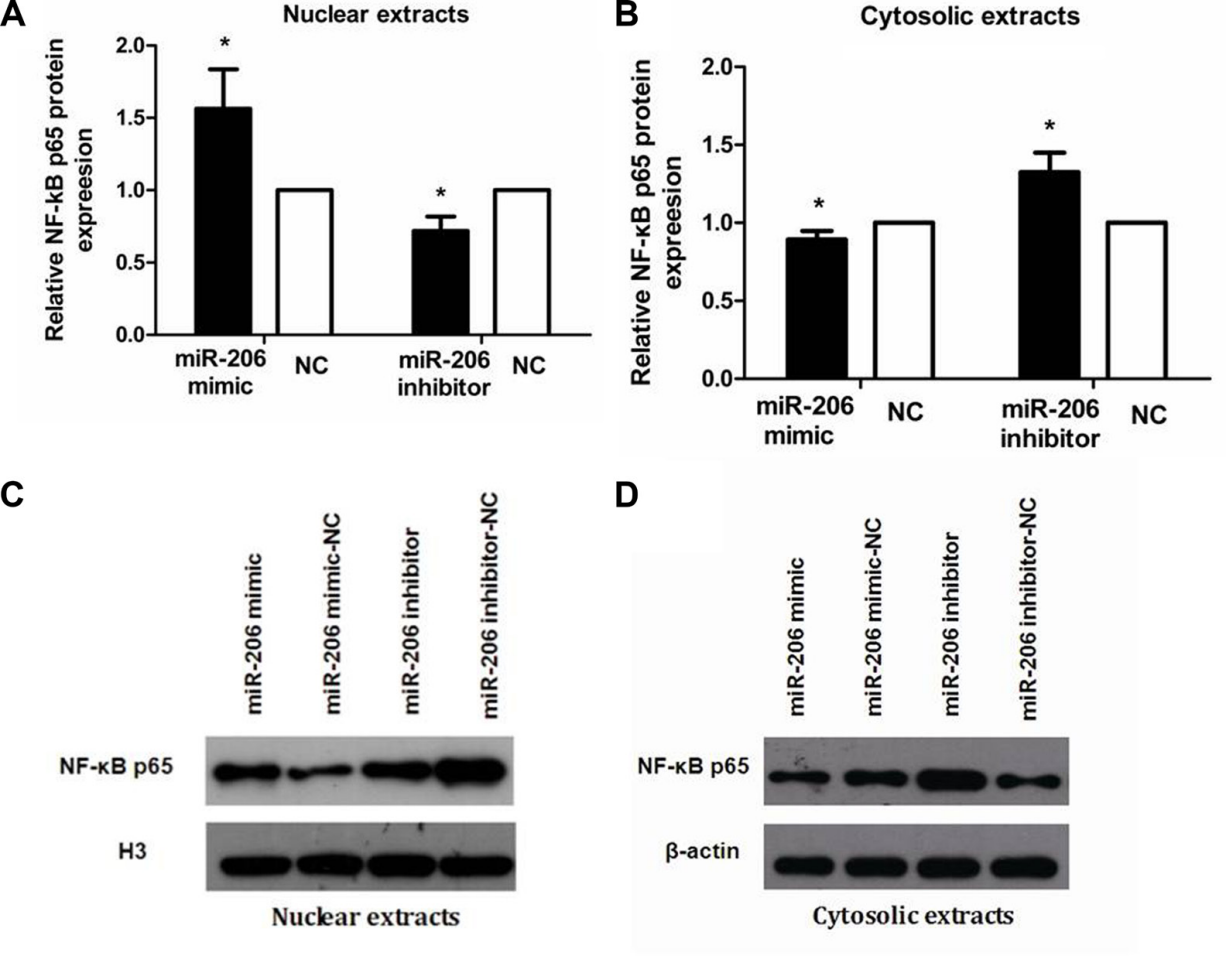
miR-206 enhanced NF-κB p65 nuclear translocation in TNF-α-treated cells (**A**, **B**) Transfection with miR-206 mimic resulted in an increase and decrease, respectively, in nuclear and cytoplasmic NF-κB p65 expression levels in TNF-α-treated cells, whereas miR-206-inhibitor transfection led to a decrease and increase, respectively, in these levels. (**C**, **D**) Representative western blotting showing NF-κB p65 expression in the indicated groups. Results are expressed as fold-change relative to NC and presented as means ± SD of 3 independent experiments. *P < 0.05, compared with the corresponding NCs.

**Figure 6:**
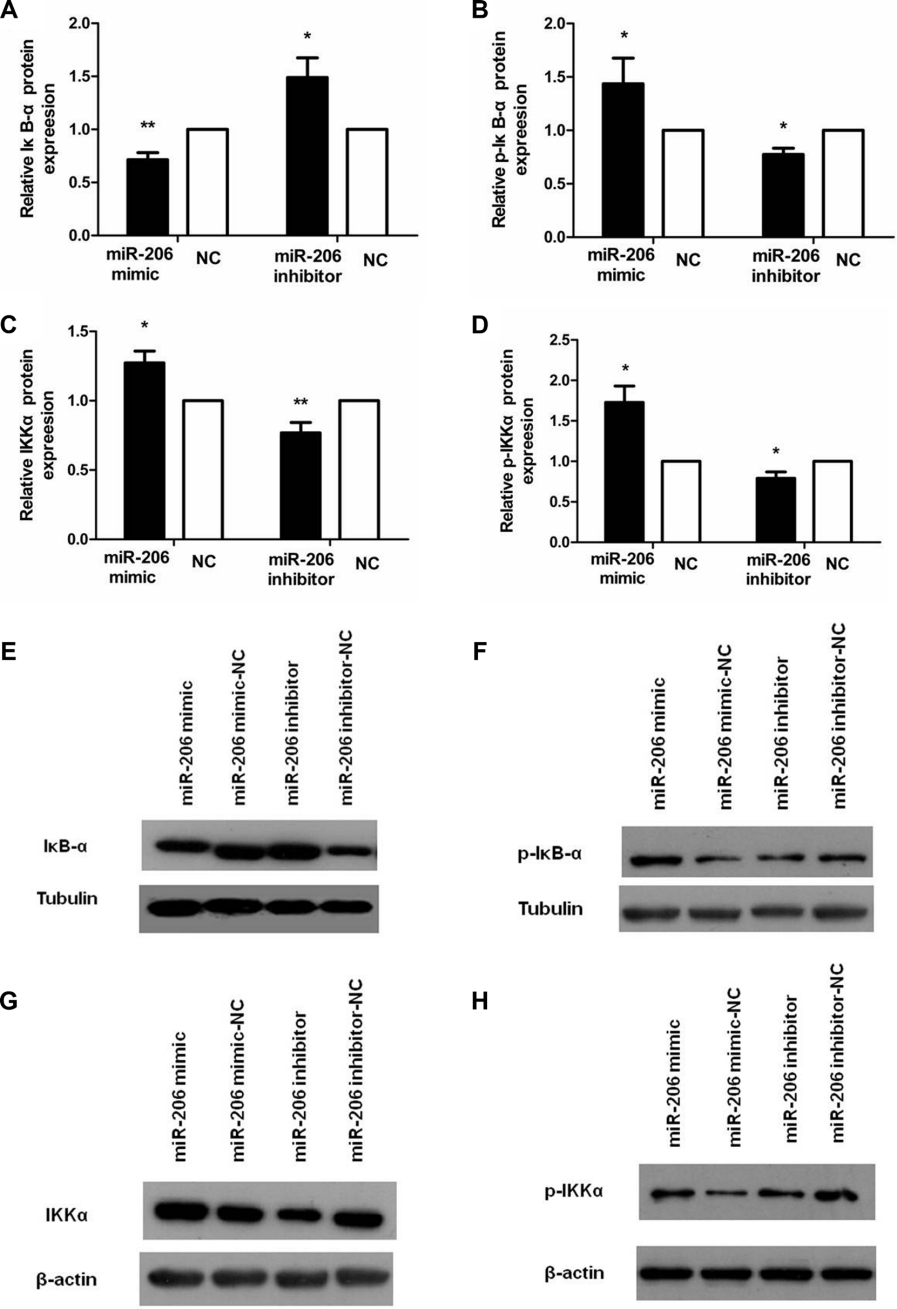
miR-206 decreased IκB-α and increase p-IκB-α, IKKα, and p-IKKα expression in TNF-α-treated cells (**A**–**D**) Transfection with miR-206 mimic resulted in a decrease in IκB-α and an increase in p-IκB-α, IKKα and p-IKKα expression levels in TNF-α-treated cells, whereas miR-206-inhibitor transfection led to an increase and decrease, respectively, in these levels. (**E**–**H**) Representative western blotting showing IκB-α, p-IκB-α, IKKα and p-IKKα expression in the indicated groups. Results are expressed as fold-change relative to NC and presented as means ± SD of 3 independent experiments. *P < 0.05 and **P < 0.01, compared with the corresponding NCs.

**Figure 7:**
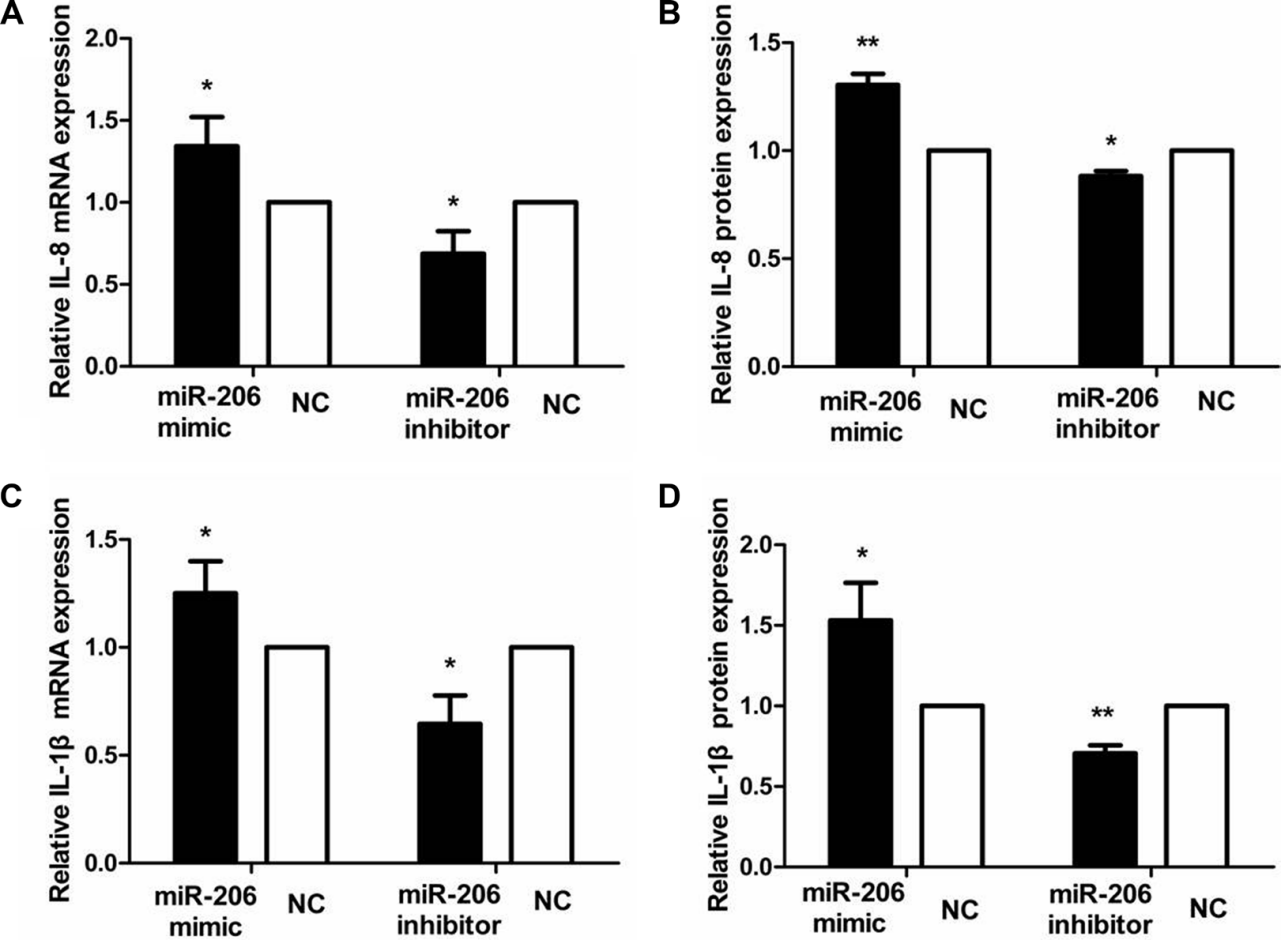
miR-206 increased IL-8/IL-1β expression in TNF-α-treated cells Transfection with miR-206 mimic and inhibitor resulted, respectively, in an increase and decrease in IL-8 and IL-1β mRNA expression and protein secretion in TNF-α-treated cells. Results are expressed as fold-change relative to NC and presented as means ± SD of 3 independent experiments. *P < 0.05 and **P < 0.01, compared with the corresponding NCs.

To examine the role of miR-206 in HT-29 cells in the context of inflammation, we measured cytoplasmic/nuclear NF-κB p65, IκB-α, p-IκB-α, IKKα, and p-IKKα protein levels and the mRNA expression and secretion of the proinflammatory cytokines IL-8 and IL-1β. HT-29 cells were transfected with miR-206 mimic/inhibitor (150 nM) for 48 or 72 h and then incubated with TNF-α (10 ng/mL) for 30 min or 24 h, following which NF-κB p65, IκB-α, p-IκB-α, IKKα, and p-IKKα protein and IL-8/IL-1β mRNA expression and secretion were assessed through western blotting, qRT-PCR, and ELISA. As compared to NC-transfected cells, miR-206-mimic-transfected HT-29 cells exhibited significantly increased and decreased nuclear and cytoplasmic NF-κB p65 expression, respectively (*P* < 0.05; Figure 5). We also observed a decrease in IκB-α and an increase in p-IκB-α, IKKα and p-IKKα expression, compared to the control groups (*P* < 0.05, Figure 6), as well as increased IL-8/IL-1β mRNA and protein expression (*P* < 0.05; Figure 7). In contrast, in miR-206-inhibitor-transfected HT-29 cells, both NF-κB p65 nuclear translocation, p-IκB-α, IKKα, p-IKKα expression and IL-8/IL-1β production were lower than in NC-transfected cells (*P* < 0.05; Figures 5, 6 and 7). These results indicate that miR-206 acts as a proinflammatory factor in the colonic inflammatory process by enhancing activation of NF-κB signalling in TNF-α-treated HT-29 cells.

### A3AR silencing can reverse the miR-206-inhibitory effect in TNF-α-induced inflammatory response

**Figure 8:**
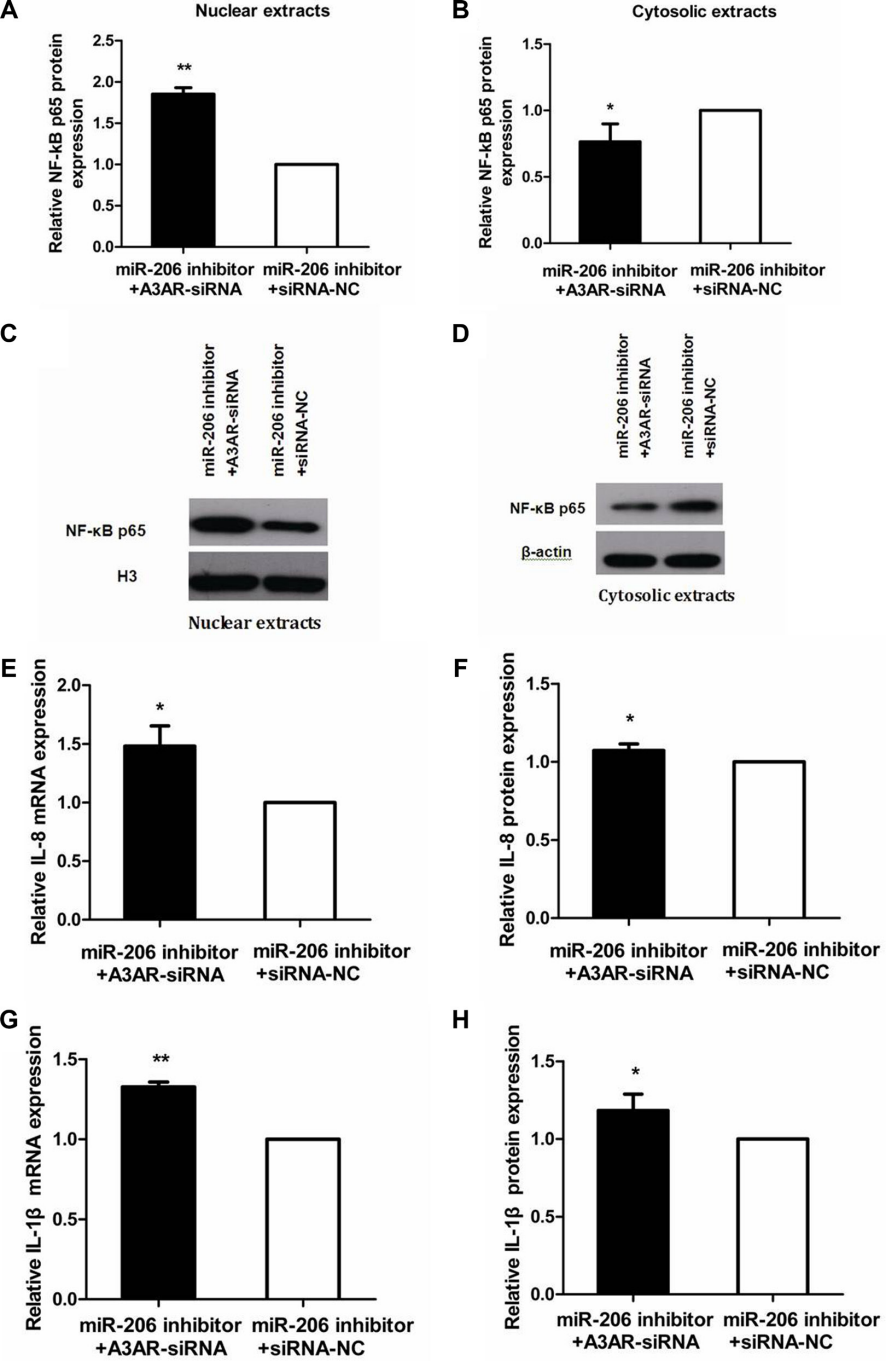
Reversal of miR-206-inhibitory effect by A3AR-siRNA (**A**, **B**) Relative to siRNA-NC transfection, A3AR-siRNA transfection abrogated the miR-206-inhibitor effect on nuclear and cytoplasmic NF-κB p65 expression in TNF-α-treated cells. (**C**, **D**) Representative western blots showing NF-κB p65 expression in the indicated groups. (**E**–**H**) Relative to siRNA-NC transfection, A3AR-siRNA transfection alleviated the miR-206-inhibitor effect on IL-8/IL-1β mRNA expression and secretion in TNF-α-treated cells. Results are expressed as fold-change relative to the corresponding NC and presented as means ± SD of 3 independent experiments. *P < 0.05; **P < 0.01.

To investigate whether the effect of miR-206 on NF-κB p65, IL-8, and IL-1β expression during the inflammatory process is exerted through A3AR, we transfected miR-206 inhibitor and A3AR-siRNA/siRNA-NC into HT-29 cells and treated the cells with TNF-α. Relative to cotransfection with siRNA-NC, cotransfection of miR-206 inhibitor with A3AR-siRNA resulted in decreased and increased cytoplasmic and nuclear NF-κB p65 protein levels, respectively, in TNF-α-treated cells (*P* < 0.05; Figure 8A–8D), and also led to increased IL-8/IL-1β mRNA expression and secretion (*P* < 0.05; Figure 8E–8H). These data indicated that A3AR-siRNA could reverse the anti-inflammatory effect of the miR-206 inhibitor, implying that miR-206 acts via A3AR to facilitate the inflammatory process.

### miR-206 increases the severity of DSS-induced colitis

**Figure 9:**
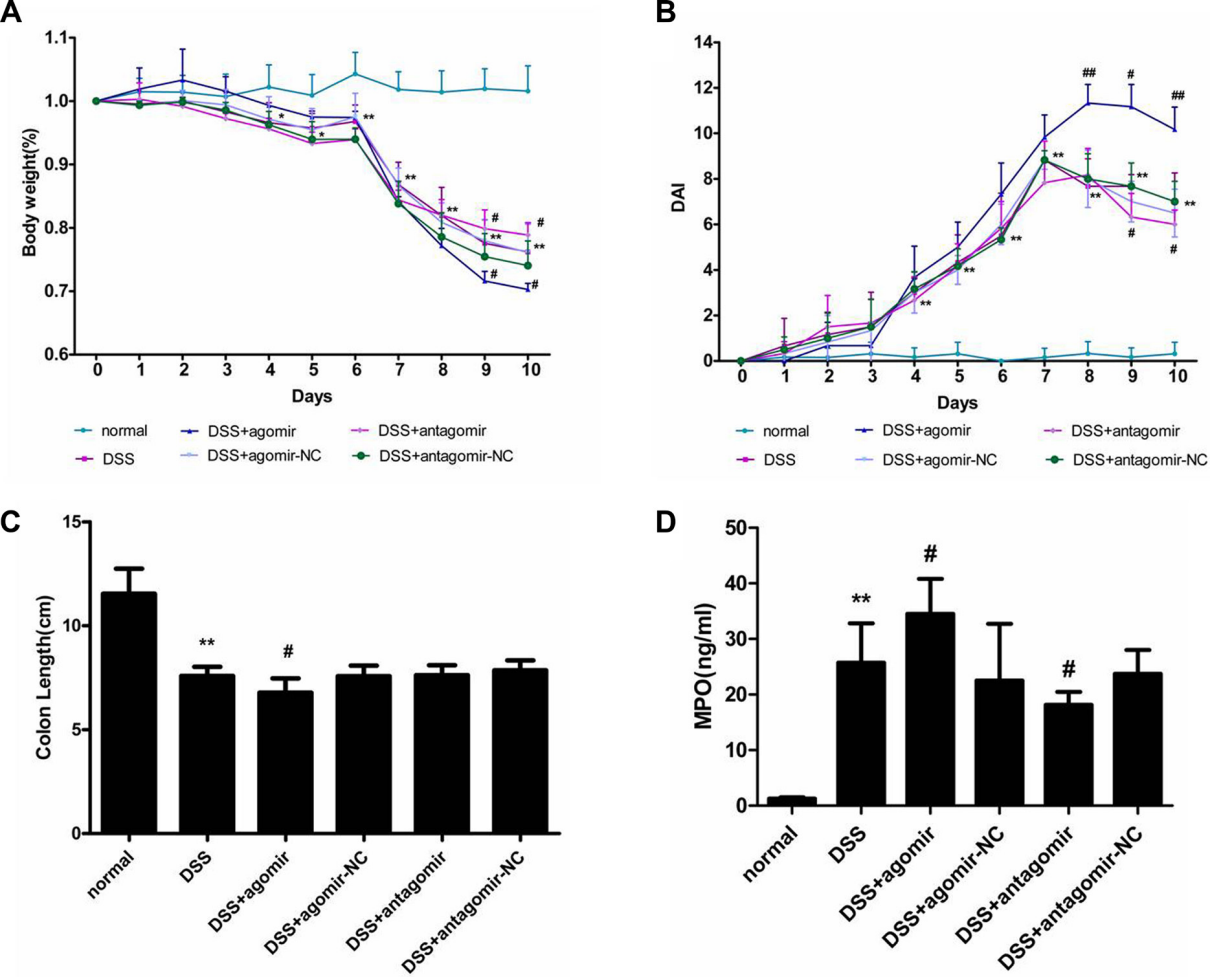
miR-206 increases the severity of DSS-induced colitis (**A**) Bodyweight was reduced in the DSS-treated group from Day 4 onwards; bodyweight reduction was worsened and improved by miR-206 agomir and antagomir administration, respectively. (**B**) DAI was increased in the DSS-treated group from Day 4 onwards; DAI increase was aggravated and improved by miR-206 agomir and antagomir administration, respectively. (**C**) DSS treatment resulted in colon shortening in mice, and miR-206-agomir administration caused further shortening. (**D**) MPO activity was significantly increased in the colon of DSS-treated mice, and was significantly increased and decreased after miR-206 agomir and antagomir treatment, respectively. All data are expressed as means ± SD. **P < 0.01, DSS-treated group versus normal group; ^#^*P* < 0.05 and ^##^*P* < 0.01, DSS+agomir/antagomir group versus corresponding NC group.

Our findings suggested that miR-206 exerts a proinflammatory effect on TNF-α-induced inflammation response in HT-29 cells by activating NF-κB signalling. Thus, we used a mouse colitis model to investigate whether miR-206 produces a similar effect *in vivo*. Oral DSS administration for 7 days caused bodyweight reduction and DAI increase relative to the control group, with the change being significant from Day 4 onwards (*P* < 0.05; Figure 9A and 9B). Moreover, the colon was shortened in the DSS group relative to controls (*P* < 0.05; Figure 9C).

DSS-treatment-induced bodyweight loss was significantly worsened or alleviated (relative to NC groups) following miR-206 agomir or antagomir administration, respectively (*P* < 0.05; Figure 9A). Furthermore, relative to NC treatments, miR-206 agomir and antagomir treatments increased and decreased, respectively, the DAI in DSS-induced colitis in mice (*P* < 0.05; Figure 9B). The colon was shorter in the miR-206-agomir-treated group than in the agomir-NC-treated group (*P* < 0.05), however, the antagomir did not significantly affect colon shortening (Figure 9C).

Myeloperoxidase (MPO) activity in the colon was significantly higher in DSS-colitis mice than that in untreated mice (*P* < 0.05). Moreover, colon MPO activity was higher in the miR-206-agomir-treated group than that in the agomir-NC-treated group (*P* < 0.05), and lower in the antagomir-treated group than that in the antagomir-NC-treated group (*P* < 0.05) (Figure 9D).

### miR-206 suppresses A3AR expression in the mouse colon

**Figure 10:**
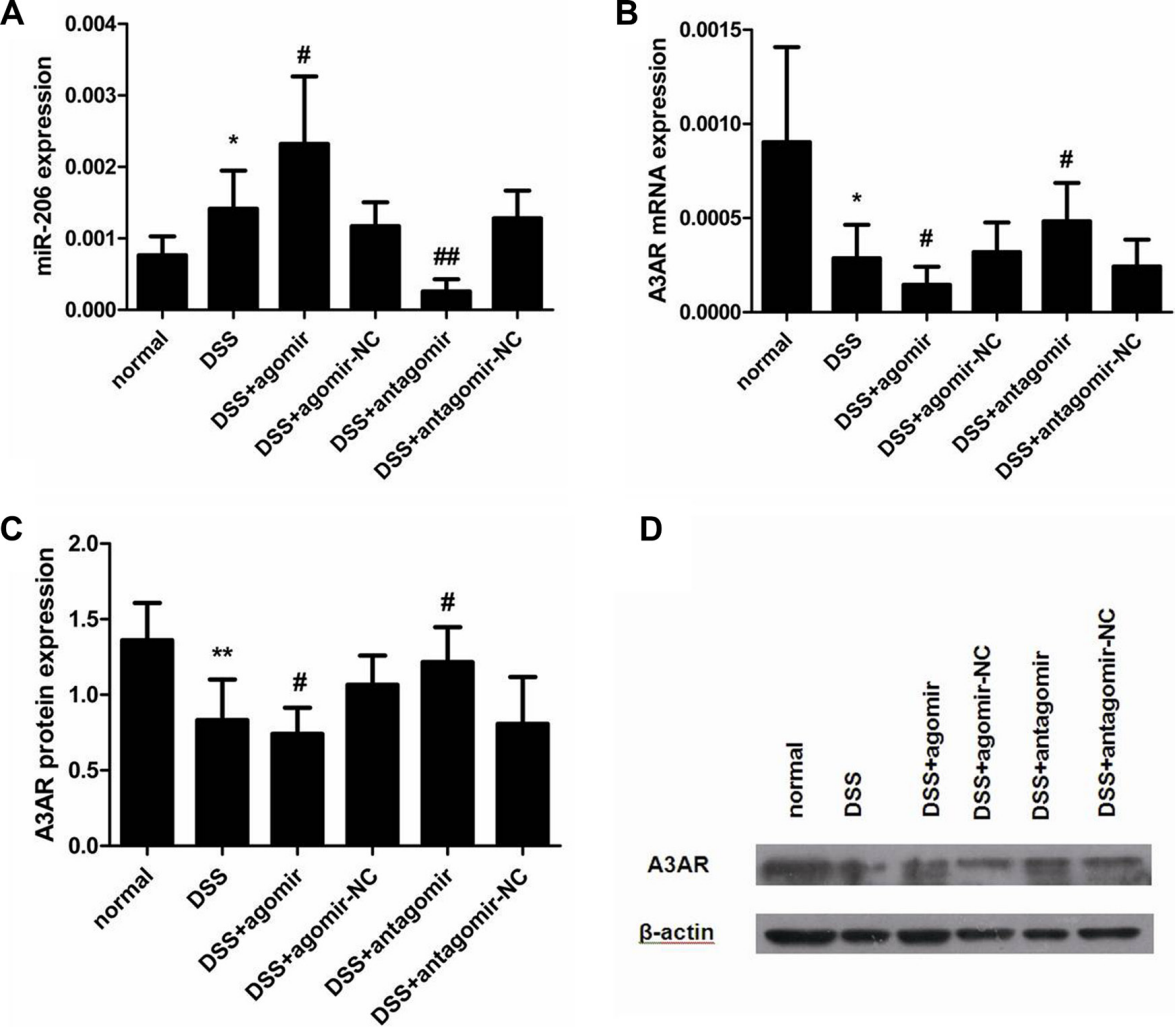
miR-206 downregulates A3AR expression in mouse colon (**A**) miR-206 expression was higher in DSS-colitis mice than in control mice, and was upregulated and downregulated in the colon of agomir- and antagomir-treated mice, respectively, as compared with corresponding NC levels. (**B**, **C**) A3AR mRNA and protein expression was significantly decreased in DSS-colitis mice, and was significantly downregulated and upregulated after miR-206 agomir and antagomir treatment, respectively (relative to corresponding NC levels). (**D**) Representative western blotting showing A3AR expression following various treatments. Results are presented as means ± SD. *P < 0.05 and **P < 0.01, compared with normal group; ^#^*P* < 0.05 and ^##^*P* < 0.01, compared with corresponding NC group.

miR-206 was highly expressed in the colon of DSS-colitis mice, whereas A3AR mRNA and protein levels were significantly decreased following DSS treatment, as compared with the control group (*P* < 0.05; Figure 10). Because miR-206 regulated A3AR expression *in vitro*, we used the miR-206 agomir and antagomir to enhance and knockdown miR-206 expression respectively, in DSS-colitis mice and thus examine miR-206 effects on A3AR expression *in vivo*. qRT-PCR results demonstrated that miR-206 expression was significantly increased and decreased, respectively, in the colon of agomir- and antagomir-treated mice as compared with levels in the corresponding NC mice (*P* < 0.05; Figure 10A). Furthermore, A3AR mRNA and protein expression in the colon was significantly lower in miR-206-agomir-treated DSS-mice than in agomir-NC-treated DSS-mice, but was higher in antagomir-treated mice than in antagomir-NC-treated mice (*P* < 0.05; Figure 10B–10D). These results suggest that miR-206 regulates A3AR expression *in vivo*.

### miR-206 stimulates NF-κB signallingpathway activation and upregulates downstream inflammatory cytokines expression in DSS-colitis mice

**Figure 11:**
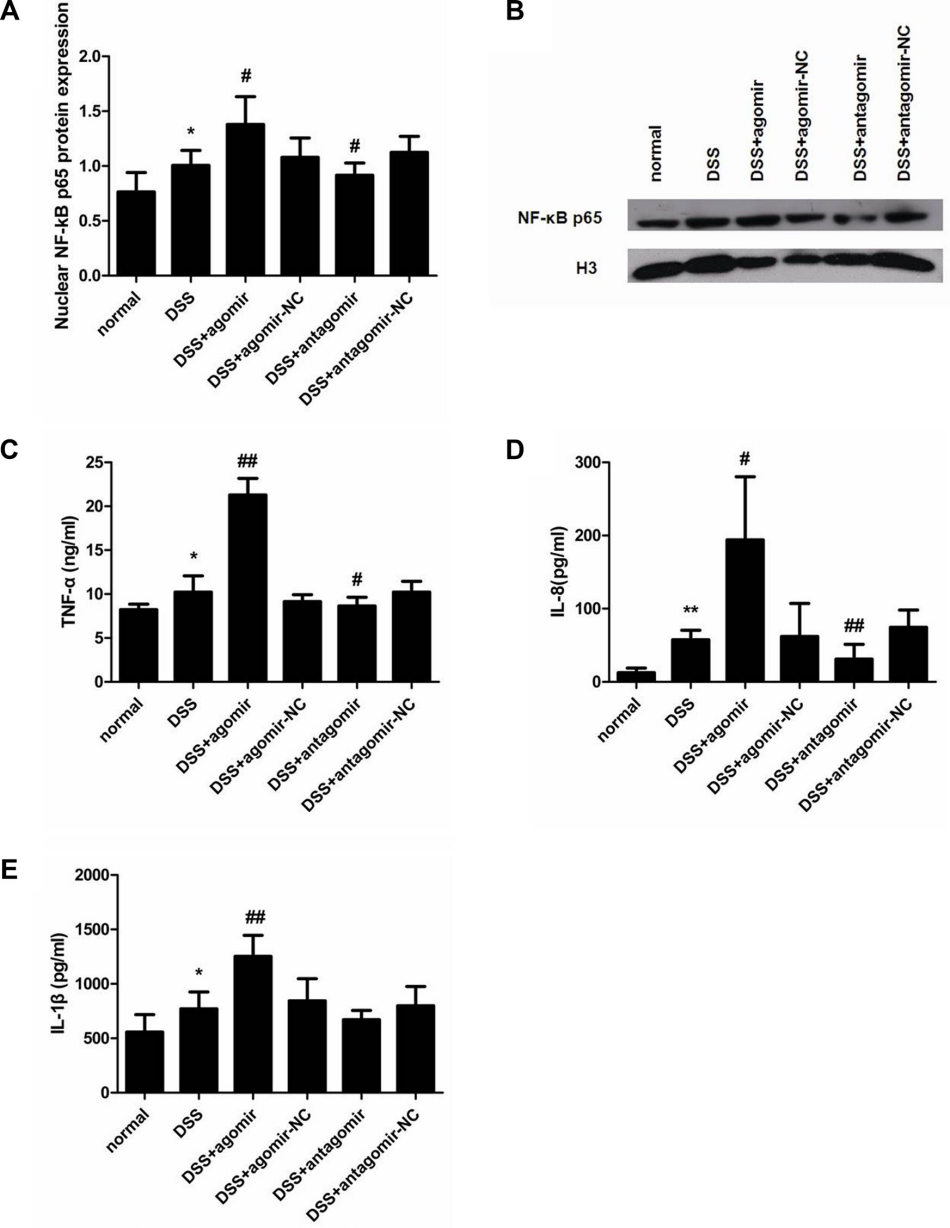
miR-206 upregulates nuclear NF-κB p65 expression and TNF-α/IL-8/IL-1β levels in mouse colon (**A**) Western-blotting analysis of nuclear NF-κB p65 expression in mouse colon. Nuclear NF-κB p65 expression was higher in DSS-colitis mice than in control mice. miR-206 agomir and antagomir treatment led to increased and decreased nuclear NF-κB p65 expression in the colon of DSS-colitis mice, respectively, as compared with corresponding NC levels. (**B**) Representative western blotting showing NF-κB p65 expression in the indicated groups. ELISA analysis of the expression of TNF-α (**C**), IL-8 (**D**), and IL-1β (**E**). TNF-α, IL-8, and IL-1β levels were significantly increased in the colon of DSS-treated mice; moreover, TNF-α, IL-8, and IL-1β expression was significantly increased after miR-206-agomir treatment, but TNF-α and IL-8 expression was decreased following miR-206-antagomir treatment (relative to corresponding NC levels). All data are expressed as means ± SD. *P < 0.05 and **P < 0.01, compared with DSS-treated group; ^#^*P* < 0.05 and ^##^*P* < 0.01, compared with corresponding NC group.

To determine whether miR-206 agomir/antagomir treatment affects cytokine levels, we measured nuclear NF-κB p65 expression and TNF-α/IL-8/IL-1β levels in the colon of mice from all groups. Relative to control levels (normal group), nuclear NF-κB p65 and TNF-α/IL-8/IL-1β levels were markedly increased in the colon of DSS-treated mice (*P* < 0.05; Figure 11). During DSS induction, miR-206-agomir treatment significantly increased (relative to agomir-NC treatment) nuclear NF-κB p65 and TNF-α/IL-8/IL-1β expression in the colon (*P* < 0.05). Conversely, miR-206-antagomir administration markedly reduced (relative to antagomir-NC treatment) the levels of these inflammatory cytokines, which had been upregulated in response to DSS treatment (*P* < 0.05) (Figure 11). These results agree with our *in vitro* findings and suggest that miR-206 enhancedNF-κB signalling and its downstream inflammatory cytokines in mice with DSS-induced colitis.

## DISCUSSION

The potential role of miRNAs in UC has been extensively investigated as the characterisation of dysregulated miRNAs in UC might facilitate an understanding of the inflammatory process and the development of UC treatments. Aberrant miRNA expression in UC including miR-206 might serve as a new disease biomarker [14]. miR-206 has been studied in several pathological processes. For example, miR-206 was shown to function as a tumour suppressor in certain tumours overexpressing miR-206 target genes [25]. In addition, miR-206 appears to act through multiple pathways to exacerbate cardiac injury [26, 27]. MiR-206 upregulation, as observed in a mouse model of Alzheimer's disease, downregulates the neuroprotective protein brain-derived neurotrophic factor, which likely contributes to disease pathogenesis [28]. Enhanced miR-206 expression in astrocytes following lipopolysaccharide (LPS) stimulation in turn led to increased inflammatory cytokine expression. Furthermore, miR-206 enhanced the LPS-induced proinflammatory response by targeting NR4A2 and activating NF-κB activity [29].

However, although miR-206 upregulation has been reported in UC, its role in colonic inflammation remains unidentified. Because miRNA-206 regulates several genes that are implicated in the progression of inflammation, investigation of miR-206 in UC might provide insights into the development of colonic inflammation.

Bioinformatics analysis to identify putative miR-206 targets in UC identified that miR-206 could bind to the 3′′-UTR of A3AR mRNA. A3AR is involved in several pathophysiological processes and has been identified as a novel anti-inflammatory target. In mice, pretreatment with the A3AR-specific agonist Cl-IB-MECA minimized LPS-induced characteristic lung inflammation [30]. Conversely, A3AR overexpression such as in rheumatoid arthritis facilitates multiple anti-inflammatory responses, consistent with those demonstrated in our previous research in human colonic epithelial cells [31]. Our previous research confirmed that A3AR activation exerts an anti-inflammatory effect in human colonic epithelial cells. Specifically, 2-Cl-IB-MECA treatment prior to TNF-α stimulation resulted in attenuated NF-κB p65 nuclear translocation, inhibited Iκ-Bα degradation, markedly reduced phosphorylated-Iκ-Bα levels, and also significantly decreased TNF-α-stimulated IL-8/IL-1β mRNA expression and secretion [18]. Moreover, we found that A3AR is expressed in murine colonic epithelia, and that selective A3AR activation produces an anti-inflammatory effect by suppressing the proinflammatory cytokine expression associated with the inhibition of NF-κB signalling pathways in murine DSS colitis *in vivo* [23].

In the current study, we first evaluated miR-206 and A3AR expression patterns in UC and normal tissues. Compared to control tissues, we found marked elevation of miR-206 expression in UC tissues but lower A3AR mRNA and protein expression, with an inverse correlation observed. IF staining of colonic tissue revealed that A3AR was mainly located on the membrane of colorectal mucosal epithelial cells, consistent with our previous reports [18, 23], and showed levels of staining in UC vs. normal tissues consistent with the western blotting results. Furthermore, *in vitro* functional-analysis results demonstrated that A3AR mRNA and protein expression was decreased and increased in HT-29 cells following transfection with miR-206 mimic and inhibitors, respectively, in a concentration-dependent manner. In addition, dual-luciferase reporter-assay results indicated that A3AR was a direct target of miR-206. These findings suggested that miR-206 downregulates A3AR expression by binding to the 3′-UTR of A3AR mRNA.

As our previous research revealed that A3AR activation exerts an anti-inflammatory effect by inhibiting NF-κB signalling pathways, we investigated the specific effect of miR-206 on NF-κB signalling and the production of downstream inflammatory cytokines. The miR-206 mimic and inhibitor enhanced or decreased NF-κB p65 nuclear translocation (indicated by increased and decreased nuclear and cytoplasmic p65 levels), p-IκB-α, IKKα, p-IKKα expression and IL-8 and IL-1β mRNA and secreted-protein levels in TNF-α-treated HT-29 cells, respectively, demonstrating that miR-206 exerted proinflammatory effects. Furthermore, A3AR silencing partially reversed the dampening effects on NF-κB signalling of the miR-206 inhibitorTherefore, we inferred that miR-206 produces proinflammatory effects at least partly by negatively regulating A3AR in TNF-α-induced inflammation in HT-29 cells.

We also obtained similar results in a colitis mouse model, wherein miR-206 increased the severity of DSS-induced colitis, measured as enhanced bodyweight loss, DAI score, colon shrinkage, and MPO activity, whereas miR-206-antagomir treatment partially ameliorated these effects. Notably, our *in vivo* results showed inversely correlated A3AR and miR-206 expression and NF-κB signalling modulation in agreement with the *in vitro* data. Thus, we concluded that miR-206 inhibited A3AR expression, activated NF-κB signalling, and promoted proinflammatory cytokine production, both *in vivo* and *in vitro*. However, the mediatingeffects of miR-206 antagomir on colon shrinkage and IL-1β expression were not statistically significant; this might have been due to incomplete blocking of miR-206 action by the antagomir, inadequate antagomir dosage, or because of the participation of additional signalling pathways *in vivo*, which represents a more complex environment than that *in vitro*. Further studies such as with miR-206-knockout mice might clarify these issues and shed additional light on the function of miR-206 *in vivo*.

In summary, our data provide evidence that miR-206 is upregulated in UC, and that miR-206 acts as a proinflammatory factor in the inflammatory response by directly suppressing A3AR expression. Thus, miR-206 is involved in UC pathogenesis and might serve as both a diagnostic and therapeutic target for UC treatment. However, given the complexity of miRNA functions under distinct circumstances, additional studies are required to clarify the role of miR-206 in UC.

## MATERIALS AND METHODS

### Human specimens

Colonic mucosa biopsies were obtained from the colon of active-UC patients (*n* = 26) and healthy people (*n* = 19) who underwent colonoscopy between March 2013 and April 2014 at The Affiliated Hospital of Guangdong Medical University, China; the institution's Medical Ethics Committee approved this study. UC diagnoses were confirmed based on history, clinical symptoms, colonoscopy, and pathological findings. The samples were embedded in paraffin for immunofluorescence analysis or snap-frozen in liquid nitrogen, and then stored at −80°C.

### Cell culture and transfection

HT-29 cells were cultured in RPMI-1640 medium (Gibco, MD, USA) supplemented with 10% foetal bovine serum (Gibco), 100 IU/mL penicillin, and 100 μg/mL streptomycin at 37°C in a 5% CO_2_ incubator. In all cases, cells were cultured in antibiotic-free medium for 24 h before the experiments. miR-206 mimic, miR-206 mimic-negative control (NC), miR-206 inhibitor, miR-206 inhibitor-NC, A3AR-siRNA, and siRNA-NC were purchased from RiboBio (Guangzhou, China). The detail sequences were listed in Table 1. HT-29 cells were seeded into 6-well plates and transfected with 50/100/150 nM mimic/mimic-NC and inhibitor/inhibitor-NC for gain-of-function and loss of-function experiments, respectively, to evaluate the effect of miR-206 on A3AR expression. HT-29 cells transfected with 150 nM mimic/mimic-NC or inhibitor/inhibitor-NC were treated with TNF-α for 30 min or 24 h respectively, and examined for nuclear/cytoplasmic NF-κB p65 levels, and IκB-α, p-IκB-α, IKKα, p-IKKα, IL-8/IL-1β levels. Lastly, analyses were performed after transfecting HT-29 cells with miR-206 inhibitor/inhibitor-NC (150 nM) and A3AR-siRNA/siRNA-NC (100 nM) and treating cells with TNF-α for 30 min or 24 h. All miRNA/siRNA transfections were performed using Lipofectamine 2000 (Invitrogen, CA, USA) in serum-free and antibiotic-free Opti-MEM I (Gibco), as per manufacturer instructions.

**Table 1:** Detail sequence of miR-206 mimic, inhibitor, agomir and antagomir, A3AR siRNA, WT and MUT A3AR

Name	Detail sequences (5′-3′)
hsa-miR-206 mimic	UGGAAUGUAAGGAAGUGUGUGG
	CCACACACUUCCUUACAUUCCA
has-miR-206 inhibitor	CCACACACUUCCUUACAUUCCA
mmu-miR-206 agomir	UGGAAUGUAAGGAAGUGUGUGG
	CCACACACUUCCUUACAUUCCA
mmu-miR-206 antagomir	CCACACACUUCCUUACAUUCCA
A3AR siRNA	GCCTACTGCTTATCTTTAC
WT A3AR	CTGAAGATTTTTTTAATTTAGTTCATAAAGTGATGCTACAACAGAA
	TAATCACCATGACAACTGGCCCACACCTCAGAGACTGATTCTGATC
	TCCCAGGAATTCTGAAGGTCCCTCTATCCTTGACAACAATCATTTG
	CAGCCAGGTAGCAACGGCAGTAGTCAGAGGAGCTATGATAGACCA
	CACCCAAGCAAGGCTGCCCTCAAATAACATCTCAAGATCTTAGTT
	CTTATGCATTCCATCAGTCAGAAGTGAAGAAGAGGTGGAGAATCT
	GGATTGGGGACCAGGAAATCACTTGTATTTTGTTAGCCAATAAAT
	TCCTAGCCAGTGTTGAATGAA
MUT A3AR	CTGAAGATTTTTTTAATTTAGTTCATAAAGTGATGCTACAACAG
	AATAATCACCATGACAACTGGCCCACACCTCAGAGACTGATTCT
	GATCTCCCAGGAATTCTGAAGGTCCCTCTATCCTTGACAACAAT
	CATTTGCAGCCAGGTAGCAACGGCAGTAGTCAGAGGAGCTATGA
	TAGACCACACCCAAGCAAGGCTGCCCTCAAATAACATCTCAAGA
	TCTTAGTTCTTATGGTAAGGTTCAGTCAGAAGTGAAGAAGAGGT
	GGAGAATCTGGATTGGGGACCAGGAAATCACTTGTATTTTGTTA
	GCCAATAAATTCCTAGCCAGTGTTGAATGAA

### Animal models

We purchased 36 male 7–8-week-old BALB/c mice (18–21 g) from Guangdong Medical Laboratory Animal Center, China. Mice were group-housed under controlled temperature (25°C) and a 12/12-h light/dark cycle and provided food and water freely. Colitis was induced in mice by administering 5% dextran sodium sulphate (DSS; MW, 40,000–50,000; MP Biomedicals, CA, USA) in drinking water for 7 consecutive days. miR-206 agomir, miR-206 agomir-NC, miR-206 antagomir and miR-206 antagomir-NC were purchased from RiboBio (Guangzhou, China). The detail sequences were listed in Table 1. Mice were randomly divided into 6 groups of 6 animals each: (1) normal, received untreated drinking water; (2) DSS, received 5%-DSS water for 7 consecutive days; (3) DSS+miR-206 agomir; (4) DSS+miR-206 agomir-NC; (5) DSS+miR-206 antagomir; and (6) DSS+miR-206 antagomir-NC. Mice in groups (3)–(6) received tail-vein injections of miRNA agomir/antagomir or their respective NCs daily for 3 consecutive days after starting 5%-DSS oral administration for 7 consecutive days; agomir/agomir-NC and antagomir/antagomir-NC doses were 20 and 200 nmol/day, respectively. On Day 14 after colitis induction, mice were sacrificed through cervical dislocation, and the colons were collected for analyses. Colon length was also measured.

### Assessment of disease severity in DSS-colitis mice

The mice were observed daily for morbidity and scored daily to assess colitis activity by using the disease-activity index (DAI, 0–12) based on weight loss, diarrhoea, and bloody faeces. We used 5 weight-loss grades (0, no loss or weight gain; 1, 1–5% loss; 2, 5–10% loss; 3, 10–20% loss; 4, 20% loss), 3 stool-consistency grades (0, normal; 2, loose; 4, diarrhoea), and 3 occult-blood grades (0, negative; 2, occult blood-positive; 4, gross bleeding) [24]. The occult-blood test (Baso Diagnostics, Inc., Zhuhai, China) was used to detect faecal occult blood in the gastrointestinal tract daily.

### Real-time quantitative reverse-transcription PCR (qRT-PCR)

We used real-time qRT-PCR to measure miR-206 and A3AR mRNA expression in UC tissues, transfected cells, and mice, and IL-8/IL-1β mRNAs in transfected cells. Transfected cells were cultured for 48 h and then total RNA was extracted using RNAiso Plus (Takara, Tokyo, Japan) and reverse-transcribed using the Mir-X miRNA First Strand Synthesis Kit (Clontech, CA, USA) for miR-206 and the PrimeScript^@^RT Master Mix (Perfect Real Time) (Takara) for A3AR/IL-8/IL-1β mRNAs, according to manufacturer protocols. As internal controls, we used U6 small-nuclear RNA for miR-206 and β-actin mRNA for A3AR/IL-8/IL-1β mRNAs. PCR was performed using SYBR^@^Premix Ex Taq^™^ II (Perfect Real Time) (Takara) in a LightCycler 480 II system (Roche Diagnostics, Indiana, USA). Table 2 lists the PCR primers (synthesised by Sangon Biotech, Shanghai, China). The mRQ 3′ primer was included in the purchased kit. The relative levels of miR-206 and A3AR/IL-8/IL-1β mRNAs were evaluated using the 2^−**Δ**Ct^ method.

**Table 2:** Gene-specific primers used for qRT-PCR

Gene	Forward sequence (5′-3′)	Reverse sequence (5′-3′)
Human miR-206	CGTGGAATGTAAGGAAGTGTGTGG	mRQ 3′ Primer
Human U6	CTCGCTTCGGCAGCACA	mRQ 3′ Primer
Human A3AR	GGCTGCCCTCAAATAACATC	CTCCACCTCTTCTTCACTTCTG
Human IL-1β	GGCAATGAGGATGACTTGTTCT	CTGTAGTGGTGGTCGGAGATTC
Human IL-8	GCAGAGGGTTGTGGAGAAGT	AACCCTACAACAGACCCACA
Human β-actin	GGCGGCAACACCATGTACCCT	AGGGGCCGGACTCGTCATACT
Mouse miR-206	TGGAATGTAAGGAAGTGTGTGG	mRQ 3′ Primer
Mouse U6	CTCGCTTCGGCAGCACA	mRQ 3′ Primer
Mouse A3AR	CGGGAGTTCAAGACAGCTAAGT	CACATTGCGACATCTGGTATCT
Mouse β-actin	CTTCTTCTTGGTATGGAATCCTG	GTAATCTCCTTCTGGATCCTGTC

### Western blotting

Transfected cells were incubated for 72 h before the western-blotting analysis. Total protein was extracted from human/mouse tissues and cells, using a lysis buffer containing 1% phenylmethylsulphonyl fluoride (Beyotime, Jiangsu, China). HT-29-cell nuclear and cytosolic extracts were collected separately using a nuclear and cytoplasmic protein-extraction kit (Sangon). Protein concentrations were quantified using a bicinchoninic acid assay kit (Beyotime). Equal amounts of protein were separated using SDS-PAGE and electroblotted onto polyvinylidene fluoride membranes (Millipore, MA, USA), which were incubated (overnight, 4°C) with primary antibodies: mouse anti-human A3AR (1:800), mouse anti-human NF-κB p65 (1:800), IκB-α (mouse anti-human, 1:400), p-IκB-α (mouse anti-human, 1:600), IKKα (mouse anti-human, 1:400), or p-IKKα (rabbit anti-human, 1:400), mouse anti-mouse A3AR (1:600), mouse anti-mouse NF-κB p65 (1:600) from Santa Cruz Biotechnology (CA, USA); mouse anti-human β-actin (1:1000), mouse anti-mouse β-actin (1:1000), tubulin (mouse anti-human, 1:1000) or rabbit anti-human histone 3 (H3) (1:500) from Beyotime; or rabbit anti-mouse H3 (1:600) fromProteintech (Wuhan, China). Membranes were washed thrice with Tris-buffered saline containing Tween 20, and then incubated (room temperature (RT), 1 h) with secondary antibodies (horseradish peroxidase-conjugated goat anti-rabbit/anti-mouse IgG, 1:1000; Beyotime). Immunoreactive bands were visualised using an enhanced chemiluminescence detection reagent (Beyotime). A3AR, cytosolic NF-κB p65 protein, IKKα and p-IKKα levels were normalised against β-actin levels, IκB-α and p-IκB-α levels were normalised against tubulin levels and nuclear NF-κB p65 levels were normalised against H3 levels. Images were analysed using Quantity One software (Bio-Rad, CA, USA).

### Immunofluorescence (IF)

IF staining for A3AR was examined in UC and normal colonic tissue. Human colonic tissue-section slides were deparaffinised in xylene and rehydrated in a graded alcohol series. After blocking endogenous peroxidase activity by incubating with hydrogen peroxide, the slides were immersed in antigen-retrieval buffer and boiled for 15 min to retrieve antigens. Subsequently, nonspecific antigens were blocked (1 h, RT) with a blocking solution (PBS containing 10% donkey serum albumin and 0.1% Triton X-100), and then the slides were incubated (overnight, 4°C) with rabbit anti-human A3AR (1:30; Santa Cruz Biotechnology), washed thrice with PBS, and incubated in the dark (1 h, RT) with secondary FITC-conjugated donkey anti-rabbit antibodies (1:100; Jackson ImmunoResearch Laboratories, PA, USA). A fluorescence mounting medium containing 4′6-diamidino-2-phenylindole (DAPI) was used to counterstain nuclei. Images were obtained using a Leica fluorescence confocal microscope.

### Dual-luciferase reporter assay

Wild-type (wt) and mutant (mut) putative miR-206-binding sites in the 3′-UTR of human A3AR mRNA, termed pmiR-A3AR-wt and pmiR-A3AR-mut, respectively, were generated through PCR-amplification and cloning into the pmiR-RB-REPORT luciferase-reporter plasmid (RiboBio). HT-29 cells were cotransfected with 500 ng of pmiR-A3AR-wt or pmiR-A3AR-mut plus 150 nM miR-206 mimic/inhibitor or their NCs by using Lipofectamine 2000. After 48-h incubation, luciferase activity was assessed using a dual-luciferase reporter-assay system (Promega, Madison, USA).

### Myeloperoxidase (MPO) activity assay

The activity of MPO, a neutrophil-infiltration indicator, was measured using an MPO ELISA kit (Boster, Wuhan, China). Colonic samples were homogenised on ice in PBS and their supernatants were collected and used in assays, per manufacturer instructions.

### ELISA

IL-8/IL-1β protein secretion from HT-29 cells and TNF-α/IL-8/IL-1β expression in the mouse colon were measured using ELISA (Sangon), per manufacturer instructions. HT-29 cell supernatants were collected after various treatments, and for the colonic samples, supernatants were collected after tissue homogenisation.

### Statistical analysis

All experiments were repeated at least thrice. Data are expressed as means ± SD. Student's *t* test was used for comparisons between 2 groups, and one-way ANOVA for comparisons of >2 groups. Correlations between miR-206 and A3AR expression were evaluated using Pearson correlation analysis. All analyses were performed using SPSS 19.0; *P* < 0.05 was considered statistically significant.
